# Assessing the Impact of Potential Confounders on Health-Related Quality of Life and Physical Activity in Patients with Chronic Kidney Disease Treated with Dialysis: A Cross-Sectional Study

**DOI:** 10.3390/healthcare13141729

**Published:** 2025-07-17

**Authors:** Georgia Paraskeva, Vasiliki Michou, Nikolaos Koutlianos, Dimitra Mameletzi, Evangelia Kouidi

**Affiliations:** Sports Medicine Laboratory, School of Physical Education and Sport Science, Aristotle University, 57001 Thessaloniki, Greece; georgialpar@gmail.com (G.P.); vasilikimichou@yahoo.gr (V.M.); koutlian@phed.auth.gr (N.K.); mamel@phed.auth.gr (D.M.)

**Keywords:** hemodialysis, health-related quality of life, physical activity, sociodemographic factors

## Abstract

**Background**: Patients with chronic kidney disease (CKD) G5 treated with dialysis (G5D) often experience reduced physical activity levels and impaired health-related quality of life (HRQoL), which are associated with poor clinical outcomes. Understanding the factors that influence these outcomes is crucial for improving patient care. This study aimed to evaluate the levels of physical activity and HRQoL and investigate the influence of potential confounding factors on these outcomes in patients with CKD G5D. **Methods**: One hundred and twenty-five patients with CKD G5D and 129 healthy controls completed a template with their general demographic and clinical information, followed by the short version of the International Physical Activity Questionnaire (IPAQ). Moreover, for patients with CKD G5D, the kidney disease-targeted version (KDQOL-SF36) was employed, whereas the healthy controls completed the standard SF-36. **Results**: A total of 59.2% of patients with CKD G5D demonstrated low physical activity levels, with a mean IPAQ score of 1163.38 MET-min/week, which was significantly lower than that of healthy controls (*p* = 0.002). Spearman’s rho correlation analysis revealed significant associations between KDQOL subscales and variables including sex, age, Charlson Comorbidity Index (CCI), hemodialysis (HD) vintage, educational level, employment status, and IPAQ activity category (*p*-values < 0.05). In the regression analyses, physical component summary (PCS) scores were significantly predicted by sex (β = 0.180, *p* = 0.036), CCI (β = 0.239, *p* = 0.045), and IPAQ total score (β = 0.316, *p* < 0.001). IPAQ scores were predicted by age (β = –0.303, *p* = 0.003), HD vintage (β = 0.275, *p* = 0.012), and PCS (β = 0.343, *p* = 0.002). **Conclusions**: The findings demonstrated a statistically significant association between physical activity and HRQoL, underscoring the importance of promoting physical activity among patients with CKD G5D. Additionally, several underexplored sociodemographic and clinical confounders were identified as significant correlates of these outcome measures.

## 1. Introduction

Patients with chronic kidney disease (CKD) stage G5 treated with dialysis (CKD G5D) often experience significantly reduced physical activity levels compared to the general population. This decline in activity can be attributed to several physiological and clinical factors associated with the progression of CKD. Uremia, caused by the accumulation of toxins due to impaired kidney function, contributes to muscle weakness, fatigue, and reduced exercise tolerance in patients with CKD. These symptoms are linked to the loss of skeletal muscle strength, resulting from both fiber atrophy and reduced fiber number. Oxidative stress is believed to play a key role, as skeletal muscles produce reactive oxygen and nitrogen species (ROS/RNS), especially during contraction, which may impair muscle function when unbalanced by antioxidant defense [1,2]. In addition, chronic inflammation [3] and oxidative stress [4] promote protein-energy waste, leading to skeletal muscle atrophy and diminished physical performance. Anemia, a common complication of CKD characterized by reduced hemoglobin levels and impaired oxygen transport, contributes to tissue hypoxia, exacerbates fatigue, and significantly limits patients’ capacity for sustained physical activity [5,6]. Furthermore, fluid overload, peripheral neuropathy, and disturbances in bone mineral metabolism are commonly observed in patients with CKD G5D and can lead to pain, discomfort, and reduced mobility, all of which contribute to physical inactivity [7,8]. These physiological impairments, combined with the burden of comorbidities and time constraints imposed by dialysis schedules, contribute to a more sedentary lifestyle in this population.

Physical activity is a critical component in maintaining health and improving the health-related quality of life (HRQoL) in patients with CKD G5D. Despite the known benefits of physical activity in promoting physical functioning, reducing fatigue, and enhancing psychological well-being, patients with CKD G5D often experience low levels of physical activity due to various barriers, including disease and comorbidities, treatment schedules, and physical limitations [9,10]. Patients with CKD G5D consistently demonstrate markedly lower levels of physical activity than healthy individuals [11]. Inactive patients frequently report lower overall HRQoL, with deficits in physical functioning, vitality, and mental health [12,13]. Studies utilizing tools like the International Physical Activity Questionnaire (IPAQ) [12,13] and the Kidney Disease Quality of Life Short Form (KDQOL-SF) [14,15] have highlighted the negative impact of inactivity on both physical and mental health outcomes in this population.

Sociodemographic factors such as age, sex, and the presence of comorbidities can significantly influence physical activity levels [16,17] and HRQoL [15,17,18] in patients with CKD G5D. For instance, older age is often associated with decreased physical activity and poorer physical functioning [19], as well as lower scores in both the Physical Component Summary (PCS) and Mental Component Summary (MCS) of the SF-36 [20]. Sex differences also play a role, with some studies showing that males tend to report better physical functioning [16,17], higher PCS scores [21], and fewer role limitations due to physical conditions than females [15,18]. The presence of comorbidities, such as diabetes, cardiovascular disease, and hypertension, can further exacerbate the limitations of physical activity [16,22], leading to a poorer overall HRQoL [18,20]. For example, patients with diabetes or cardiovascular conditions may report significantly lower physical functioning and higher symptom burden as measured by the KDQOL-SF [22,23].

Additionally, sociodemographic factors such as marital status, employment, and educational level, which have only been slightly investigated in patients with CKD G5D, may also influence physical activity and HRQoL. Marital and employment statuses may affect a patient’s social support network and psychological well-being [16,24], while higher educational attainment may be associated with better health literacy and greater engagement in health-promoting behaviors [14,16,25]. Although often underexplored, these factors may provide critical insights for improving care strategies tailored to the specific needs of patients with CKD G5D. This highlights the importance of considering a broader range of variables when designing interventions aimed at enhancing physical activity and overall HRQoL in this vulnerable patient group. The current study focused on a Greek cohort of patients with CKD G5D, a population that has received limited attention in prior research. The aim of this study was twofold: first, to compare physical activity levels and HRQoL between patients with CKD G5D and healthy controls, and second, to examine the extent to which sociodemographic factors (such as sex, employment, marital status, and educational level) and clinical characteristics (including comorbidity burden, hemodialysis vintage, and type of dialysis access) predict physical activity and HRQoL among patients with CKD G5D. It was hypothesized that patients with CKD G5D would exhibit significantly lower physical activity levels and HRQoL than healthy individuals and that selected sociodemographic and clinical variables would serve as significant predictors of both outcomes.

Although physical activity and HRQoL have received increasing attention in patients with CKD, current research provides limited insight into the combined effects of sociodemographic and clinical determinants, especially in individuals with CKD G5D. This limitation hampers the development of targeted, evidence-based interventions aimed at improving the outcomes of this population. Although validated tools such as the IPAQ and KDQOL-SF are widely used, few studies have adopted an integrative framework that simultaneously accounts for a broad range of potential confounders. The present study addresses this gap by incorporating both underexplored sociodemographic characteristics and clinically relevant variables into a unified analytical model. This approach offers a novel contribution to the literature, particularly in the context of dialysis-dependent CKD populations.

In this study, confounding factors were operationally defined as variables that may exert independent effects on both physical activity and HRQoL, thereby obscuring or distorting the true associations between these outcomes. Specifically, this study examined the influence of sociodemographic variables, such as sex, marital status, educational attainment, and employment status, alongside clinical indicators, including dialysis vintage, comorbidity burden as measured by the Charlson Comorbidity Index (CCI), and type of dialysis access. The primary objective of this study was to compare physical activity levels and HRQoL between patients with CKD G5D and a healthy control group. The secondary objective was to evaluate the extent to which the identified sociodemographic and clinical confounding variables predicted physical activity and HRQoL among patients with CKD G5D. It was hypothesized that (1) individuals with CKD G5D would exhibit significantly lower physical activity levels and HRQoL scores than healthy controls, and (2) selected sociodemographic and clinical variables would serve as significant predictors of these outcomes within the CKD G5D cohort.

## 2. Materials and Methods

### 2.1. Study Design and Settings

This cross-sectional study was conducted between March and December 2024. Hemodialysis (HD) units across Greece were invited to participate in the study through an overview of its aims and methodology. Data were collected during routine clinical visits. This study aimed to evaluate the levels of physical activity and HRQoL and examine the influence of potential confounders in patients with CKD G5D compared to healthy controls.

### 2.2. Participants

Patients with CKD G5D were recruited from dialysis units in various cities across Greece. All participants were screened for eligibility based on the established inclusion and exclusion criteria. The inclusion criteria for patients with CKD G5D included being older than 18 years of age, being ambulatory, and having a confirmed diagnosis of CKD that necessitated maintenance hemodialysis treatment. The exclusion criteria were as follows: younger than 18 years of age, severe musculoskeletal disorders that could limit mobility or exercise, and prior participation in similar research studies. Additionally, age- and sex-matched healthy individuals were included in the control group.

### 2.3. Sample Size Estimation

Our sample size was determined using a power analysis for a one-way ANOVA test with a significance level (α) set at 0.05. To achieve 80% power, a total of 220 participants were required. More precisely, for a sample size of 220, the calculated power was 0.895, assuming a Type I error rate (α = 0.05) of 5% and a Type II error rate (β = 0.105) of 10.5% (since power = 1 − β, and 1 − 0.895 = 0.105). The initially planned allocation for this calculation was 110 participants with CKD G5D and 110 healthy controls. In our study, we successfully recruited and evaluated 254 participants, exceeding the calculated requirement. The participants were assigned to two unequal groups: 125 participants with CKD G5D and 129 healthy controls.

### 2.4. Reduction of Potential Bias

Several steps were taken to minimize the potential sources of bias in this cross-sectional study. To reduce selection bias, patients were consecutively recruited from multiple hemodialysis units across different geographic locations in Greece, ensuring a diverse and representative sample. Age- and sex-matched healthy individuals were included as controls to account for confounding variables. Information bias was minimized using validated and standardized instruments (IPAQ, SF-36, and KDQOL-SF), and all data were collected by trained personnel following a consistent protocol. Recall bias was mitigated by using short recall periods in the IPAQ and self-reported questionnaires. Finally, to address potential confounding, multiple linear regression analyses were conducted to adjust for key variables, such as sex, dialysis vintage, and comorbidity burden.

### 2.5. Data Collection and Measurements

Those who met the predefined inclusion criteria were asked to complete a demographic and clinical information questionnaire. Subsequently, two standardized instruments were administered: (1) the short form of the IPAQ and (2) the kidney disease-targeted version (KDQOL-SF36) in patients with CKD G5D and the standard SF-36 in healthy controls. A comorbidity index was used for both groups.

#### 2.5.1. IPAQ

The IPAQ, a validated and widely used instrument for assessing physical activity across populations, was employed in this study using the validated Greek version [26]. The short form of IPAQ consists of nine items and estimates total physical activity in metabolic equivalent task minutes per week (MET-min/week). Based on the total MET-minutes per week, participants were classified into one of three physical activity categories: low, moderate, or high.

#### 2.5.2. SF-36 and KDQOL-SF

The Greek-validated standard SF-36 questionnaire was used to assess self-perceived HRQoL in healthy participants [13]. This questionnaire consists of 36 items distributed across eight subscales, yielding two summary scores: the PCS and MCS. The subscales evaluate physical health (including physical functioning, role limitations due to physical health, bodily pain, and general health) and mental health (comprising vitality, social functioning, role limitations due to emotional problems, and mental health), with higher scores indicating a better QoL. For patients with CKD G5D, the Greek-validated version of the KSQOL-SF was used [18,27]. This version includes eight SF-36 subscales and 11 CKD-specific scales addressing symptoms, the effects and burden of kidney disease, work status, social interaction, cognitive function, sleep, social support, dialysis staff encouragement, and patient satisfaction.

#### 2.5.3. Charlson Comorbidity Index (CCI)

The CCI was utilized. The CCI is a globally validated tool that predicts mortality by quantifying comorbidities across various disease groups, including cancer, renal disease, and cardiovascular disease [28]. While few studies have assessed its validity in patients with CKD G5D, this population exhibits a high mortality and comorbidity burden [29].

### 2.6. Data Verification

To ensure data completeness and quality, all questionnaires were administered in person during scheduled clinical visits by trained members of the research team. Immediately after completing the questionnaire, each form was reviewed for completeness and consistency. Any missing or unclear responses were clarified directly with the participants in real time. This protocol enabled the accurate and comprehensive collection of all demographic, clinical, and patient-reported data. As a result, no missing data were recorded for the IPAQ, SF-36, KDQOL-SF, or other key study variables.

### 2.7. Confounding Factors

The identification and control of potential confounding variables were guided by both theoretical considerations and empirical evidence from prior studies investigating the determinants of physical activity and HRQoL in patients with CKD. The variables considered as potential confounders included age, sex, educational attainment, employment status, comorbidity burden (quantified using the CCI [30,31]), dialysis vintage, and vascular access type, categorized as arteriovenous fistula (AVF), arteriovenous graft (AVG), or central venous catheter (CVC) [32]. These factors were selected based on their established or hypothesized influence on both exposure (e.g., physical activity) and outcome (e.g., HRQoL) measures. To statistically adjust for these potential sources of bias, we used multiple linear regression models in which physical activity (IPAQ total score) and HRQoL (SF-36 PCS score) served as dependent variables. This approach enabled the estimation of independent associations between key predictors and outcomes while accounting for the influence of measured confounders.

### 2.8. Ethical Considerations

The study protocol received ethical approval from the Research Ethics Committee of the Aristotle University of Thessaloniki (Protocol No. 196/2024). Written informed consent was obtained from all participants in line with the Declaration of Helsinki (2013), and data confidentiality was ensured in compliance with the GDPR (EU Regulation 196/2024).

### 2.9. Statistical Analysis

Statistical analyses were performed using IBM SPSS Statistics for Windows, Version 28.0 (IBM Corp., Armonk, NY, USA). Descriptive statistics were used to summarize the data: categorical variables were reported as frequencies (n) and percentages (%), while continuous variables were presented as means (M) ± standard deviation (SD). No missing data were identified for the IPAQ, KDQOL-SF, or the SF-36 instruments. Group comparisons for continuous outcomes (e.g., age, PCS, and IPAQ scores) were performed using independent *t*-tests for dichotomous variables (e.g., previous transplantation: Yes/No) and one-way ANOVA for variables with three or more categories (e.g., marital status). Post-hoc comparisons following ANOVA were conducted using Bonferroni correction. Effect sizes were calculated based on the type of statistical tests performed. For comparisons between two groups (e.g., sex), independent *t*-tests were used, with Cohen’s d reported as the effect size (interpreted as small ≥ 0.2, medium ≥ 0.5, and large ≥ 0.8). For comparisons involving three or more groups (e.g., marital, employment, and educational status), one-way ANOVAs were conducted, and effect sizes were expressed using partial eta squared (η^2^), with thresholds for small (≥0.01), medium (≥0.06), and large (≥0.14) effect sizes. Spearman’s rho rank correlation coefficient was used to evaluate the correlation between patients’ SF-36 subscales scores and clinical/demographic data. Finally, to assess the effects of potential confounders (e.g., sex and HD vintage) on the PCS and IPAQ scores, multiple linear regression analyses were performed. A *p*-value < 0.05 was considered statistically significant.

## 3. Results

### 3.1. Demographic and Clinical Characteristics

One hundred and twenty-five patients with CKD G5D and 129 healthy controls participated in this study. The participants’ demographic and clinical characteristics are presented in Table 1.

### 3.2. IPAQ, KDQOL-SF, and SF-36 Results

**Figure 1:**
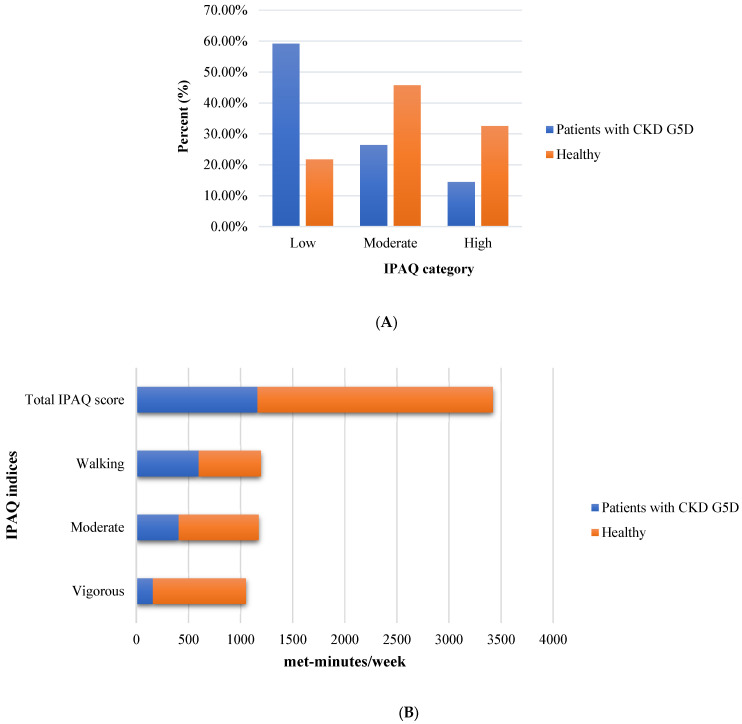
Comparison of physical activity levels assessed by the International Physical Activity Questionnaire (IPAQ) among patients with chronic kidney disease (CKD) G5 treated with dialysis (G5D) and healthy controls. (**A**) Distribution of participants across the IPAQ physical activity categories (low, moderate, and high), presented as percentages for each group. A statistically significant difference was observed between the groups in the distribution of activity categories (*p* < 0.05). (**B**) Mean weekly physical activity levels by IPAQ subcomponents, including vigorous activity, moderate activity, walking, and the total IPAQ score, expressed in MET-minutes/week for both groups. Patients with CKD G5D showed significantly lower levels of physical activity for vigorous, moderate, and total scores but not for walking when compared to healthy controls.

**Figure 2:**
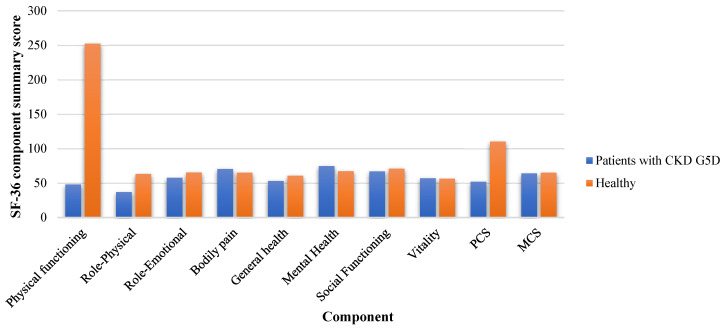
Comparison of health-related quality of life (HRQoL) scores measured using the SF-36 questionnaire among patients with chronic kidney disease (CKD) G5 treated with dialysis (G5D) and healthy controls. The bar chart presents the mean scores for each SF-36 subscale and the summary component scores, including the Physical Component Summary (PCS) and Mental Component Summary (MCS). Statistically significant differences were observed between the groups for Physical Functioning, Role-Physical, Bodily Pain, and PCS, with healthy individuals consistently reporting higher scores in these physical health domains (*p* < 0.05). These findings indicate impaired physical aspects of HRQoL among patients undergoing hemodialysis compared to healthy controls.

The participants’ IPAQ and KDQOL-SF/SF-36 results are presented in Supplementary Tables S1 and S2, respectively. The results indicated that patients with CKD G5D had significantly lower levels of physical activity, as measured by the IPAQ category (*p* < 0.001) and total IPAQ score (met-minutes/week) (*p* = 0.002) (Figure 1). Regarding the differences between the groups in the SF-36, the findings showed that patients with CKD G5D had significantly lower scores in physical functioning (*p* < 0.001), role physical (*p* < 0.001), bodily pain (*p* = 0.031), and PCS (*p* = 0.028) (Figure 2).

### 3.3. Group Comparisons Based on PCS and IPAQ Total Scores in Patients with CKD G5D

The findings presented in Table 2 indicate that the patients’ PCS scores varied significantly across several factors. Notably, both sex [t(123) = 2.408, *p* = 0.018, Cohen’s d = 0.45] and marital status [F(4, 120) = 4.693, *p* = 0.001, η^2^ = 0.13] showed statistically significant differences with moderate effect sizes. Specifically, male patients had higher PCS scores than female patients, and divorced individuals reported significantly higher PCS scores than widows and widowers. Employment status also revealed a significant correlation with PCS scores [F(4, 120) = 2.764, *p* = 0.031, η^2^ = 0.08], with unemployed patients demonstrating higher PCS scores than university students did. However, the category of physical activity measured by the IPAQ exhibited the strongest correlation with PCS scores [F(4, 120) = 11.231, *p* < 0.001, η^2^ = 0.46], suggesting that higher levels of physical activity are strongly associated with better physical health outcomes (Table 2).

Likewise, Table 3 demonstrates significant differences in the IPAQ total scores based on key demographic and clinical factors. Sex [t(123) = 2.556, *p* = 0.001, Cohen’s d = 0.14], marital status [F(4, 120) = 6.190, *p* < 0.001, η^2^ = 0.18], educational status [F(4, 120) = 4.465, *p* = 0.013, η^2^ = 0.07], and dialysis access type [t(123) = 2.851, *p* = 0.005, Cohen’s d = 0.54] were significantly associated with physical activity levels. Although the effect sizes for sex and education were small to moderate, the large effect size for dialysis access underscores its substantial influence on activity levels. Similarly, male patients, divorced individuals, and those with higher education levels exhibited greater physical activity levels (Table 3).

### 3.4. Spearman’s Rho Rank Correlation Between KDQOL-SF Subscales Scores

Spearman’s rho rank correlation analysis of the KDQOL-SF subscales in patients with CKD G5D revealed statistically significant low to moderate associations (Table 4). The strongest positive correlation was between sexual function and the IPAQ activity category (r = 0.425, *p* < 0.001). Conversely, the strongest negative correlation was found between physical functioning and HD vintage (r = −0.467, *p* < 0.001).

### 3.5. Multiple Regression Results

Multiple regression analysis revealed a relationship between the total score of IPAQ and various independent variables. The results showed that age (*p* = 0.003), HD vintage (*p* = 0.012), and PCS score (*p* = 0.002) significantly contributed to the model (Table 5). Specifically, the results revealed that 28.2% of the variability observed in the total score of IPAQ was explained by the regression model (R^2^ = 0.282, F = 4.386, *p* < 0.001). Additionally, by using the PCS score as a subordinate variable, the analysis showed that sex (*p* = 0.036), CCI (*p* = 0.045), total IPAQ score (*p* < 0.001), and IPAQ activity category (*p* = 0.034) had a statistically significant contribution to the model (Table 6), which explained 27.8% of the total variance (R^2^ = 0.278, F = 4.137, *p* = 0.001).

## 4. Discussion

This study aimed to evaluate the levels of physical activity and HRQoL in Greek patients diagnosed with CKD G5D. The results indicated that 59.2% of the patients exhibited low levels of physical activity, with a mean total IPAQ score of 1163.38 MET-minutes per week, which was significantly lower than that observed in the healthy control group. Furthermore, the PCS score derived from the SF-36 was more than twice as high in the control group, reflecting markedly poorer physical health among patients with CKD G5D. Conversely, no statistically significant difference was observed in the MCS scores between the two groups.

In our study examining potential confounding factors related to physical activity in patients with CKD G5D, we identified several variables significantly associated with the total IPAQ scores, including sex, marital status, educational level, and type of dialysis access. Male patients demonstrated significantly higher physical activity levels than female patients, although the effect size was small (*p* = 0.001, Cohen’s d = 0.14). Marital status had a more pronounced association (*p* < 0.001, η^2^ = 0.18), with divorced patients reporting the highest levels of physical activity and widowed individuals reporting the lowest. Educational status was also significantly associated with IPAQ scores (*p* = 0.013, η^2^ = 0.07), with higher activity levels observed among individuals with higher education, reflecting a moderate effect size. The strongest association was found between the type of dialysis access (*p* = 0.005, Cohen’s d = 0.54). Patients remaining with AVF or AVG access reported significantly higher levels of physical activity than those with CVC access, indicating a significant and clinically meaningful effect. These findings indicate that both sociodemographic and treatment-related factors significantly influence physical activity in the Greek CKD G5D population, making them effective targets for both behavioral and clinical intervention. These findings are consistent with those of previous studies. Older individuals and women often exhibit lower levels of physical activity, possibly due to increased physical limitations or different health behaviors [16]. Recently, a study by Koźma-Śmiechowicz et al. [33] found that regular physical activity significantly predicts all-cause mortality in patients undergoing HD. Those who engage in frequent exercise are associated with improved survival rates. In addition, while some studies have reported that the male sex may be linked to reduced activity levels [10], others have found that female patients are less likely to meet physical activity recommendations [17,34], as confirmed by our findings. Higher educational attainment is consistently associated with increased participation in physical activity, likely due to improved health literacy and motivation. In contrast, the influence of marital status on physical activity remains ambiguous, with inconsistent findings in the literature and a need for further research [23]. However, factors such as small sample size and differing categorizations of educational, employment, and marital status have been linked to no statistically significant correlations between physical activity and these socioeconomic factors [35]. Therefore, the absence of statistically significant associations between certain variables and physical activity levels in patients undergoing hemodialysis may not necessarily reflect a true lack of relationship but could instead be attributed to statistical limitations, such as insufficient power, measurement variability, or residual confounding.

Our study also found a statistically significant association between hemodialysis (HD) vintage and total physical activity (IPAQ score; *p* = 0.012) in multiple regression analysis. However, comorbidities assessed using the CCI did not show a significant relationship. Supporting our findings, Kim et al. [36] reported that HD patients with a vintage of six months or more had physical activity levels 60–70% lower than those of healthy individuals. Moreover, Michou et al. [10] found that patients on HD for 2–5 years were twice as likely to have low physical activity levels. Evidence regarding comorbidities remains inconclusive. While some studies have suggested that a higher comorbidity burden may not significantly reduce activity levels [10], others have reported negative associations between multiple comorbidities, including diabetes mellitus, hypertension, polycystic kidney disease, and cardiovascular disorders, and reduced physical activity or low-intensity exercise [19,22,23]. Moreover, HD patients with comorbidities experience significantly higher levels of psychological distress and fatigue, resulting in lower physical activity levels. However, engaging in regular weekly physical activity is associated with reduced severity of fatigue and less interference caused by fatigue [37]. These findings suggest the relevance of these factors in understanding HRQoL and designing targeted interventions for patients with CKD G5D.

Furthermore, the present study revealed statistically significant correlations between various KDQOL-SF subscales and demographic and clinical variables, reinforcing the multifactorial nature of HRQoL in patients with CKD G5D. Age was negatively associated with sexual (*p* = 0.030), cognitive (*p* = 0.019), and role-physical (*p* = 0.019) functions. These associations, although of small to moderate magnitude, suggest that aging may independently contribute to reductions in both the physical and mental components of HRQoL. These findings are consistent with prior research reporting inverse associations between age and the PCS score [15] and with evidence showing that the physical health score was lower in older adults (β: −2.5, 95% CI: −4.8 to −0.1) than in adults aged 20–59 years [21]. Recent evidence by Naseef et al. [20] similarly demonstrated significantly lower MCS and PCS scores among HD patients aged ≥ 40 years (*p* < 0.001). In the same age group, although Király et al. [38] observed that HD patients had lower PCS scores than those receiving peritoneal dialysis, but there was no statistically significant difference in MCS scores between the two groups. Additionally, Kontodimopoulos and Niakas [18] reported that age was negatively associated with all KDQOL-SF subscales in a Greek CKD G5D cohort.

In addition to age, sex, and HD vintage, HD vintage also emerged as a relevant predictor. Sex was associated with bodily pain (*p* = 0.019), whereas HD vintage was significantly correlated with physical functioning (*p* < 0.001) and vitality (*p* = 0.014). Although the effect sizes were modest, the consistent direction and statistical significance of these findings support the inclusion of these variables in the predictive models. Our results indicated that male patients had higher PCS scores than female patients, which may reflect cultural or role-related differences in physical functioning or symptom perception. Several studies have reported similar sex disparities in KDQOL-SF subscales, with females consistently demonstrating lower scores. For instance, Floria et al. [39] reported a statistically significant difference between males and females, with females exhibiting lower overall scores. In addition, females scored significantly lower on the subscales of physical functioning, symptoms, and the effects of kidney disease. Conversely, other studies have found no statistically significant associations between sex and KDQOL-SF outcomes [15,20,40]. Regarding HD vintage, patients undergoing dialysis for less than 36 months demonstrated better scores in subscales such as physical functioning, energy, and general health perception. In contrast, longer dialysis durations were linked to lower scores in the physical and symptom-related domains but paradoxically associated with higher scores in the subscales reflecting disease burden and emotional well-being [15,40]. A recent study found no significant correlation between the PCS score (*p* = 0.72) and the MCS score (*p* = 0.13) of HD patients and the frequency of HD sessions per week. However, PCS scores decreased with increasing age. In contrast, the MCS score was not significantly correlated with advancing age in HD patients [41]. These findings suggest that both sex and treatment duration should be considered when evaluating HRQoL in patients with CKD G5D.

Employment status was significantly associated with HRQoL outcomes. Unemployed patients reported a higher perceived burden (*p* = 0.014) and lower quality of social interaction (*p* = 0.007). These findings suggest small-to-moderate effects that could have psychosocial implications. Employment may not only represent an economic factor but also serve as an indicator of physical and social functioning in older adults. Similar to our results, Bodesova et al. [24] reported that HD patients who were employed during their first year showed significantly higher scores in physical functioning and mental health than those who were not employed. This indicates that employment may serve as a protective factor for the HRQoL. Furthermore, Joshi et al. [42] found that employed patients scored significantly higher in the environmental domain and general health perception than their unemployed counterparts did. However, other studies have not found significant associations between employment status and health-related quality-of-life outcomes [20]. Although the evidence is somewhat diverse, it is widely acknowledged that patients with CKD G5D face substantial barriers to maintaining employment, often due to the time commitments of dialysis and the physical limitations that accompany disease progression.

Regarding the association between educational status and KDQOL-SF subscales, our study found that educational status was correlated with physical function (*p* = 0.032) and role-physical (*p* = 0.018) subscales. Although the effect sizes were moderate, they supported the hypothesis that education enhances health literacy and facilitates self-management. Naseef et al. [20], reported that participants with a bachelor’s degree or higher had significantly higher mean scores in both the MCS (53.51 ± 20.63, *p* = 0.003) and the PCS (47.21 ± 19.15, *p* < 0.001) compared to those with lower levels of education. Likewise, Porter et al. [14] found that patients with CKD G5D who held a college degree exhibited significantly higher scores on both the PCS and MCS of the SF-36 than those with lower educational attainment (*p* < 0.005). In line with these findings, Lee and Jeon [25] observed that CKD G5D patients with more than 16 years of education had significantly higher PCS scores than those with 9 years or fewer years of education (49.4 ± 7.3 vs. 44.4 ± 10.5, *p* < 0.05). However, the MCS scores were comparable between the two groups (51.9 ± 9.2 vs. 51.8 ± 11.4; *p* > 0.05). Machaca-Choque et al. [21] found that HD patients with higher education levels reported higher MCS scores than those with only primary education. However, no significant correlation was found between education level and the PCS scores. In addition, our study revealed that the CCI was correlated with the role physical (*p* < 0.001), general health (*p* = 0.027), and PCS (*p* = 0.001). Notably, patients with lower CCI scores exhibited significantly higher PCS scores, indicating better perceived physical health among those with fewer comorbidities. The literature has shown that comorbidities are associated with significantly lower scores in SF-36 subscales, such as role physical, role emotional, and work status [20]. Interestingly, HD patients with comorbid hypertension and diabetes mellitus had significantly lower PCS scores than those without these conditions [21].

Finally, our study showed that the total IPAQ score and IPAQ activity categories were significantly correlated with most KDQOL-SF subscales. Higher physical activity levels were strongly associated with higher PCS scores. These findings suggest a positive association between regular physical activity and both physical and mental aspects of health-related quality of life in Greek patients with CKD G5D. In agreement, Hu et al. [43] showed that higher activity levels and regular exercise were significantly associated with improved QoL. Fukushima et al. [13] conducted a study examining the association between IPAQ and SF-36 outcomes in patients with CKD, reporting that 61.9% of participants were classified as physically active. Furthermore, physically active individuals have significantly higher HRQoL scores than those with insufficient activity levels [13]. Wu et al. [12] found that patients with CKD G5D who engaged in moderate-intensity physical activity reported higher SF-36 scores compared to those who participated in low-intensity activity (*p* < 0.001). Additionally, among patients with CKD G5D who had comorbidities, those who performed moderate-intensity activity demonstrated significantly better QoL (*p* < 0.001). This suggests that physical activity may help mitigate the decline in HRQoL associated with comorbidities [12]. Increasing physical activity among HD patients is crucial for enhancing their QoL, as studies have shown that HD patients exhibit significantly lower levels of physical activity and QoL than individuals receiving peritoneal dialysis [38].

In summary, this study has both strengths and limitations. A key strength of this study is the identification of marital status, employment status, and educational level as sociodemographic variables associated with physical activity levels and HRQoL in patients with CKD G5D, which have been relatively understudied in this population. Additionally, the observed associations between higher physical activity levels and multiple HRQoL domains underscore the potential relevance of lifestyle behaviors in this clinical context. The use of validated disease-specific instruments (KDQOL-SF and IPAQ), along with the inclusion of an age- and sex-matched healthy control group, further strengthens the methodological rigor of the study. However, several limitations must be acknowledged. First, the cross-sectional design precludes any inference of causality between physical activity, HRQoL, and sociodemographic and clinical variables. Therefore, the reported associations should be interpreted as correlational. Second, although the sample was drawn from multiple dialysis units across Greece, the findings may have limited generalizability to other geographic or healthcare settings. Third, reliance on self-reported questionnaires introduces the potential for recall and social desirability biases, which may affect the accuracy of responses. Moreover, the absence of objective physical activity measurements (e.g., accelerometry or use of pedometers) may limit the precision of activity level estimates. Fourth, the observational design of the study did not include an intervention component, thereby restricting conclusions regarding the effect of structured physical activity programs on HRQoL. Finally, some subgroup analyses, especially those related to marital status (such as being divorced or in a relationship) and type of dialysis access (e.g., CVC versus AVF/AVG), were conducted with very small sample sizes. These limited subgroup sizes lower the statistical power, VTE risk of both Type I and Type II errors, and diminish the reliability and generalizability of the associated findings. Therefore, the results of these comparisons should be interpreted cautiously and viewed as exploratory rather than definitive.

## 5. Conclusions

The present study assessed physical activity levels and HRQoL while considering various potential confounding factors in patients with CKD G5D. The findings revealed a significant association between physical activity and multiple dimensions of QoL, underscoring the importance of promoting physical activity in this population. Additionally, this study identified key yet often overlooked confounders that significantly affected both physical activity and QoL outcomes in the Greek population with CKD G5D. These insights highlight the necessity of personalized interventions that consider sociodemographic factors to improve patient well-being. Future research should adopt longitudinal or interventional study designs to establish causal relationships and evaluate the effectiveness of tailored physical activity programs. Moreover, incorporating objective measurements of physical activity and a wider range of psychosocial variables could provide a more comprehensive understanding of the factors influencing HRQoL in patients with CKD G5D.

## Figures and Tables

**Table 1:** Participants’ demographic and clinical characteristics.

	Patients with CKD G5D (N = 125)	Healthy (N = 129)	*p*-Value
Sex			
Male	72 (57.6%)	71 (55.0%)	*p* = 0.145
Female	53 (42.4%)	58 (44.9%)	*p* = 0.373
Age (years)	62.92 ± 11.96	61.91 ± 10.38	*p* = 0.334
BMI (kg/cm^2^)	26.57 ± 4.16	26.84 ± 5.02	
Ht (%)	35.95 ± 2.87	42.67 ± 4.89	*p* < 0.001
Hb (g/dL)	11.66 ± 0.76	12.93 ± 0.68	*p* = 0.033
Marital status			
Married	84 (67.2%)	79 (61.2%)	*p* = 0.442
Unmarried	20 (16.0%)	25 (19.3%)	*p* = 0.466
Divorced	2 (1.6%)	0 (0.0%)	*p* = 0.555
Widow/widower	17 (13.6%)	20 (15.5%)	*p* = 0.337
In relationship	2 (1.6%)	5 (3.8%)	*p* = 0.120
Employment status			
Unemployed	9 (7.2%)	12 (9.3%)	*p* = 0.136
Employed	17 (13.6%)	13 (10.1%)	*p* = 0.219
Household	19 (15.2%)	20 (15.5%)	*p* = 0.672
Retirees	79 (63.2%)	84 (65.1%)	*p* = 0.458
University student	2 (1.6%)	0 (0.0%)	*p* = 0.125
Educational status			
Primary education	32 (25.6%)	29 (22.4%)	*p* = 0.512
Secondary education	82 (65.6%)	86 (66.6%)	*p* = 0.215
Higher education	11 (8.8%)	14 (10.8%)	*p* = 0.322
Type of Dialysis Unit			
Private	92 (73.6%)	-	
Public	33 (26.4%)	-	
HD vintage (months)	52.20 ± 37.04	-	
Dialysis access			
AVF or AVG	84 (67.2%)	-	
CVC	41 (32.8%)	-	
Previous transplantation			
Yes	15 (12.0%)	-	
No	110 (88.0%)	-	
CCI	7.90 ± 0.93	2.41 ± 0.87	*p* < 0.001 *
Comorbidities			
Hypertension	34 (27.2%)	30 (23.2%)	*p* = 0.477
Diabetes mellitus	43 (34.4%)	7 (5.4%)	*p* < 0.001 *
Cardiovascular disease	16 (12.8%)	11 (8.5%)	*p* = 0.289
Dyslipidemia	5 (4.0%)	1 (0.7%)	*p* = 0.237
Osteoporosis	1 (0.8%)	1 (0.7%)	*p* = 1.000
COPD	1 (0.8%)	0 (0.0%)	*p* = 0.672
Secondary hyperthyroidism	2 (1.6%)	0 (0.0%)	*p* = 0.109
Other	35 (28.0%)	24 (18.6%)	*p* = 0.050

Note: Data are expressed as mean ± SD and frequency (percent). CKD: chronic kidney disease; G5D: CKD G5 treated with dialysis; ΒΜΙ: Body Mass Index; Ht: Hematocrit; Hb: Hemoglobin; HD: Hemodialysis; AVF: Arteriovenous fistula; AVG: Arteriovenous graft; CVC: Central venous catheter; CCI: Charlson Comorbidity Index; COPD: Chronic Obstructive Pulmonary Disease. * Statistically significant at *p* < 0.05.

**Table 2:** Correlation of PCS scores with clinical and demographic variables and physical activity category in patients with CKD G5D.

		PCS Score			
		Mean ± SD	95% CI	Effect Size (Cohen’s d/Partial η^2^)	*p*-Value
Sex	(a) Male	55.47 ± 22.99	50.36/60.58	0.45 ^d^	0.018 *
	(b) Female	45.86 ± 18.21	40.39/51.33		
Marital status	(a) Married	53.62 ± 21.56	48.94/58.30	0.13 ^η2^	0.001 *
	(b) Unmarried	55.93 ± 18.96	47.05/64.81		
	(c) Divorced	84.73 ± 9.72	−2.98/171.73		
	(d) Widow/widower	34.53 ± 14.63	27.01/42.06		
	(e) In relationship	61.38 ± 45.76	−350.27/473.03		
Employment status	(a) Unemployed	65.55 ± 17.70	51.94/79.16	0.08 ^η2^	0.031 *
	(b) Employed	63.46 ± 21.48	52.41/74.50		
	(c) Household	50.74 ± 17.91	42.11/59.37		
	(d) Retirees	48.38 ± 22.18	43.41/53.35		
	(e) University student	46.25 ± 10.60	−48.90/141.40		
Educational status	(a) Primary education	48.66 ± 21.62	40.87/56.46	0.02 ^η2^	0.346
	(b) Secondary education	52.28 ± 21.33	47.59/56.97		
	(c) Higher education	59.72 ± 25.77	42.41/77.04		
Dialysis access	(a) AVF or AVG	53.75 ± 21.99	48.98/58.52	0.24 ^d^	0.203
	(b) CVC	48.44 ± 21.28	41.72/55.16		
Previous transplantation	(a) Yes	43.13 ± 16.89	33.77/52.49	0.42 ^d^	0.093
	(b) No	53.22 ± 22.19	49.02/57.41		
IPAQ category	(a) Low	45.71 ± 21.02	40.84/50.58	0.46 ^η2^	<0.001 *
	(b) Moderate	56.52 ± 18.31	50.02/63.01		
	(c) High	69.61 ± 20.11	59.61/79.62		

Note: Associations between clinical and demographic variables and the PCS score (measured by the SF-36) were assessed using independent *t*-tests or one-way ANOVA. For two-group comparisons (e.g., sex), independent *t*-tests were performed, with Cohen’s *d* reported as the effect size. For comparisons involving three or more groups (e.g., marital, employment, and educational status), one-way ANOVA was used, and partial eta squared (η^2^) was reported as the effect size. SD: standard deviation; ᵈ: Cohen’s *d*; η^2^: partial eta squared; AVF: Arteriovenous fistula; AVG: Arteriovenous graft; CVC: Central venous catheter; PCS: Physical Component Summary; IPAQ: International Physical Activity Questionnaire. * *p* < 0.05 indicates statistical significance.

**Table 3:** Correlation of total physical activity (IPAQ MET-min/week) with clinical and demographic variables in patients with CKD G5D.

		IPAQ Score (met-minutes/week)			
		Mean ± SD	95% CI	Effect Size (Cohen’s d/Partial η^2^)	*p*-Value
Sex	(a) Male	1226.47 ± 928.08	924.40/1528.54	0.14 ^d^	0.001 *
	(b) Female	951.23 ± 765.66	740.14/1162.32		
Marital status	(a) Married	1066.99 ± 1073.55	834.01/1299.97	0.18 ^η2^	<0.001 *
	(b) Unmarried	1516.20 ± 1352.79	883.07/2149.32		
	(c) Divorced	4109.00 ± 3081.57	−23,577.82/31,795.82		
	(d) Widow/widower	663.41 ± 647.13	330.68/622.45		
	(e) In relationship	2988.00 ± 2486.18	−19,349.50/25,325.50		
Employment status	(a) Unemployed	1681.00 ± 1119.89	820.17/2541.82	0.03 ^η2^	0.382
	(b) Employed	1500.47 ± 1217.12	874.68/2126.26		
	(c) Household	939.30 ± 570.47	655.61/1222.99		
	(d) Retirees	1097.65 ± 1001 ± 77	799.58/1395.72		
	(e) University student	582.00 ± 323.07	−6813.01/7977.01		
Educational status	(a) Primary education	720.43 ± 638.81	309.85/1131.02	0.07 ^η2^	0.013 *
	(b) Secondary education	1238.57 ± 1197.63	975.42/1501.72		
	(c) Higher education	1891.45 ± 1240.23	1058.25/2724.65		
Dialysis access	(a) AVF or AVG	1374.64 ± 1350.12	1081.64/1667.63	0.54 ^d^	0.005 *
	(b) CVC	730.57 ± 715.08	498.55/962.59		
Previous transplantation	(a) Yes	1143.10 ± 753.49	725.82/1560.37	0.20 ^d^	0.946
	(b) No	1166.15 ± 1072.08	925.76/1406.54		

Note: Associations between clinical and demographic variables and the total IPAQ score (MET-min/week) in patients with chronic kidney disease (CKD) G5 treated with dialysis (G5D) were assessed using independent *t*-tests or one-way ANOVA, as appropriate. For two-group comparisons (e.g., sex), independent *t*-tests were performed, and Cohen’s d was calculated as the effect size measure. For comparisons involving three or more groups (e.g., marital, employment, and educational status), one-way ANOVA was used, and partial eta squared (η^2^) was reported as the effect size. SD: standard deviation; AVF: Arteriovenous fistula; AVG: Arteriovenous graft; CVC: Central venous catheter; IPAQ: International Physical Activity Questionnaire; ᵈ: Cohen’s d; η^2^: partial eta squared. * *p* < 0.05 indicates statistical significance.

**Table 4:** Correlation of SF-36 subscale scores with clinical and demographic variables in patients with CKD G5D.

		Spearman’s rho Rank Correlation Coefficient						
	Sex	Age	CCI	HD Vintage	EmploymentStatus	Educational Status	Total IPAQ Score (met-minutes/week)	IPAQ Activity Category
Kidney disease targeted scales								
Symptom/problems	−0.027	−0.169	0.012	0.092	−0.081	0.111	0.264 **	0.414 **
Effect of kidney disease	−0.046	−0.134	0.151	0.068	0.011	0.114	0.072	0.275 **
Burden of kidney disease	0.096	−0.016	0.102	0.038	−0.220 *	0.059	0.054	0.278 **
Work status	−0.135	−0.108	0.001	0.007	0.038	−0.140	0.031	0.062
Quality of social interaction	−0.009	0.009	0.075	−0.017	−0.239 **	0.021	0.034	0.291 **
Cognitive function	0.143	−0.210 *	0.046	0.007	−0.084	0.152	0.098	0.354 **
Sexual function	0.117	−0.233 *	0.075	0.046	−0.064	0.093	0.163	0.425 **
Sleep	−0.047	−0.080	0.161	0.101	−0.091	0.094	0.084	0.277 **
Social support	0.147	0.025	−0.061	0.102	−0.019	0.018	0.055	0.195 *
Dialysis staff encouragement	0.060	−0.010	0.000	−0.137	−0.105	−0.136	0.010	0.087
Patient satisfaction	0.024	−0.18	0.076	0.128	0.000	−0.046	0.028	−0.002
SF-36 domain/component summary score								
Physical functioning	−0.039	−0.104	0.108	−0.467 **	−0.072	0.192 *	0.353 **	0.405 **
Role-Physical	0.027	−0.210 **	0.255 **	0.100	0.055	0.211 **	0.295 **	0.270 **
Role-Emotional	0.106	−0.084	−0.027	−0.167	−0.008	0.062	0.191 *	0.209 *
Bodily pain	−0.216 **	−0.148	0.069	−0.137	−0.149	0.034	0.019	0.213 **
General health	−0.019	−0.017	0.209 *	−0.077	0.042	0.011	0.087	0.149
Mental Health	0.065	0.076	0.167	−0.042	−0.006	−0.003	0.086	0.116
Social Functioning	0.076	−0.082	0.103	−0.026	−0.099	0.098	0.140	0.169
Vitality	−0.010	−0.103	0.043	−0.319 **	−0.020	0.133	0.149	0.257 **
PCS	−0.052	−0.201 *	0.264 **	−0.140	−0.028	0.160	0.290 **	0.384 **
MCS	0.080	−0.108	0.058	−0.022	−0.037	0.112	0.197 *	0.256 **

Note: The table presents correlations between kidney disease–targeted scales of the SF-36, the SF-36 domain and component summary scores, and relevant clinical and demographic variables in patients with CKD G5D. ** Correlation is significant at the 0.01 level (2-tailed). * Correlation is significant at the 0.05 level (2-tailed). CCI: Charlson Comorbidity Index; PCS: Physical Component Summary; MCS: Mental Component Summary.

**Table 5:** Multiple linear regression analysis predicting the total IPAQ score (MET-min/week) in patients with CKD G5D.

	Unstandardized Coefficients		Standardized Coefficients	*t*-Test	*p*-Value	95% Confidence Interval for B	
	B	Std. Error	Beta			Lower Bound	Upper Bound
(Constant)	2691.894	1296.661		2.076	0.040	123.694	5260.093
Sex	185.562	226.732	0.069	0.818	0.415	−263.508	634.633
Age (years)	−30.885	10.050	−0.303	−3.073	0.003 *	−50.791	−10.979
Ht (%)	61.136	36.651	−0.144	−1.668	0.098	−133.748	11.475
BMI (Kg/m^2^)	−11.949	24.498	−0.041	−0.488	0.627	−60.470	36.572
Employment status	−62.484	97.970	−0.054	−0.638	0.525	−256.527	131.559
Educational status	72.708	92.235	0.074	0.788	0.432	−109.976	255.391
HD vintage	15.351	6.049	0.275	2.538	0.012 *	3.370	27.332
Previous Transplantation	3.655	334.389	0.001	0.011	0.991	−658.705	666.016
CCI	12.749	115.198	0.010	0.111	0.912	−215.479	240.978
PCS	19.160	6.157	0.343	3.112	0.002 *	6.971	31.350
MCS	0.121	6.205	0.002	0.019	0.985	−12.163	12.404
R^2^ = 0.282, F = 4.386, *p* < 0.001							

Note: This table presents the results of a multiple linear regression analysis predicting the total International Physical Activity Questionnaire (IPAQ) score, measured in MET-minutes per week, among patients with chronic kidney disease (CKD) G5 treated with dialysis (G5D). The analysis included unstandardized and standardized coefficients, standard errors, *t*-tests, *p*-values, and 95% confidence intervals for each predictor variable. The variables included were demographic and clinical characteristics such as sex, age, hematocrit (Ht), body mass index (BMI), employment status, educational status, hemodialysis (HD) vintage, previous transplantation, Charlson Comorbidity Index (CCI), Physical Component Summary (PCS), and Mental Component Summary (MCS) scores. * *p* < 0.05 indicates statistical significance.

**Table 6:** Multiple linear regression analysis predicts the PCS score in patients with CKD G5D.

	Unstandardized Coefficients		Standardized Coefficients	*t*-Test	*p*-Value	95% Confidence Interval for B	
	B	Std. Error	Beta			Lower Bound	Upper Bound
(Constant)	24.297	16.404		1.044	0.146	−21.116	59.709
Sex	8.165	3.850	0.180	2.121	0.036 *	0.540	15.789
Age (years)	−0.036	0.083	−0.038	−0.431	0.668	−0.199	0.128
Ht (%)	−0.090	0.651	−0.012	−0.138	0.891	−1.378	1.199
BMI (Kg/m^2^)	−0.100	0.450	−0.019	−0.222	0.825	−0.990	0.791
Employment status	−0.330	1.314	−0.022	−0.251	0.802	−2.932	2.272
Educational status	1.321	1.871	0.062	0.706	0.482	−2.385	5.028
HD vintage	0.017	0.051	0.028	.328	0.744	−0.084	0.117
Previous Transplantation	8.403	5.915	0.126	1.421	0.158	−3.312	20.118
CCI	8.544	4.192	0.239	2.038	0.045 *	0.179	16.908
Total IPAQ (met-minutes/week) score	0.006	0.002	0.316	3.618	<0.001 *	0.003	0.009
IPAQ category	8.592	4.012	0.289	2.142	0.034 *	0.649	16.536
R^2^ = 0.278, F = 4.137, *p* = 0.001							

Note: This table presents the results of a multiple linear regression analysis predicting the Physical Component Summary (PCS) score among patients with chronic kidney disease (CKD) G5 treated with dialysis (G5D). The analysis included unstandardized and standardized coefficients, standard errors, *t*-tests, *p*-values, and 95% confidence intervals for each predictor variable. Significant predictors (*p* < 0.05) are marked with an asterisk (*). The variables included were demographic and clinical characteristics such as sex, age, hematocrit (Ht), body mass index (BMI), employment status, educational status, hemodialysis (HD) vintage, previous transplantation, Charlson Comorbidity Index (CCI), International Physical Activity Questionnaire (IPAQ) score, measured in MET-minutes per week, and IPAQ category. * *p* < 0.05 indicates statistical significance.

## Data Availability

The data presented in this study are available upon request from the corresponding author. The data are not publicly available due to ethical restrictions.

## Supplementary Materials

The following supporting information can be downloaded at: https://www.mdpi.com/article/10.3390/healthcare13141729/s1, Table S1: Comparison of physical activity levels (IPAQ categories and MET-min/week indices) between patients with CKD G5D and healthy controls; Table S2: Comparison of health-related quality of life (HRQoL) scores (KDQOL-SF and SF-36 components) between patients with CKD G5D and healthy controls.

## Abbreviations

The following abbreviations are used in this manuscript:

AVF	Arteriovenous Fistula
AVG	Arteriovenous Graft
BMI	Body Mass Index
CCI	Charlson Comorbidity Index
CKD	Chronic Kidney Disease
CKD G5D	Chronic Kidney Disease Stage G5 on Dialysis
COPD	Chronic Obstructive Pulmonary Disease
CVC	Central Venous Catheter
GDPR	General Data Protection Regulation
Hb	Hemoglobin
HD	Hemodialysis
HRQoL	Health-Related Quality of Life
Ht	Hematocrit
IPAQ	International Physical Activity Questionnaire
KDQOL-SF	Kidney Disease Quality of Life – Short Form
MCS	Mental Component Summary
MET	Metabolic Equivalent of Task
PCS	Physical Component Summary
QoL	Quality of Life
SF-36	Short Form 36 Health Survey
SD	Standard Deviation
