# Supplementary material for: Assessing the Impact of Potential Confounders on Health-Related Quality of Life and Physical Activity in Patients with Chronic Kidney Disease Treated with Dialysis: A Cross-Sectional Study
Source: Healthcare (Basel). 2025 Jul 17;13(14):1729. doi: 10.3390/healthcare13141729 (PMC12294399; doi:10.3390/healthcare13141729)
Supplement: Supplementary file 1 [file healthcare-13-01729-s001.zip › healthcare-3743519-supplementary.pdf]

Table S1. Comparison of physical activity levels (IPAQ categories and MET-min/week indices) between patients with CKD G5D and healthy controls.

IPAQ category	Patients with CKD G5D (N=125)		Healthy (N=129)		Patients with CKD G5D vs. Healthy
	n (%)	95% CI	n (%)	95% CI (lower/upper bound)	p-value
Low	74 (59.2%)		28 (21.7%)		p<0.001*
Moderate	33 (26.4%)		59 (45.7%)		p<0.001*
High	18 (14.4%)		42 (32.5%)		p<0.001*
**IPAQ indices (met-minutes/week)**	**Mean±SD**		**Mean±SD**		
Vigorous	159.04±102.39	70.10/247.97	892.71±304. 13	839.73/945.69	p<0.001*
Moderate	406.56±404.74	324.28/488.83	767.06±605.97	661.49/872.63	p=0.002*
Walking	597.78±496.15	509.95/685.62	598.27±591.04	495.30/701.24	p=0.242
Total IPAQ score	1163.38±1019.26	947.53/1379.23	2258.03±1501.14	1996.51/2519.55	p=0.002*

Note: Data are presented as frequencies (percentages) for categorical variables and as means ± standard deviation (SD) for continuous variables. The table compares physical activity levels between patients with stage G5 chronic kidney disease treated with dialysis (CKD G5D) and healthy controls. Physical activity was assessed using the International Physical Activity Questionnaire (IPAQ), which classifies activities into three categories—low, moderate, and high—and quantifies activity levels in MET-minutes per week (metabolic equivalents). The IPAQ indices include vigorous activity, moderate activity, walking, and total physical activity. Confidence intervals (CI) indicate the range of plausible values for each group. Statistically significant differences ($p < 0.05$) were observed in most activity categories and indices, with patients with CKD G5D generally reporting lower physical activity levels compared to healthy controls.

*Significant at the level of p<0.05.

Table S2. Comparison of health-related quality of life (HRQoL) scores (KDQOL-SF and SF-36 components) between patients with CKD G5D and healthy controls.

	Patients with CKD G5D (N=125)		Healthy (N=129)		Patients with CKD G5D vs. Healthy
	**Mean±SD**	**95% (CI)**	**Mean±SD**	**95% CI (lower/upper bound)**	**p-value**
**Kidney disease targeted scales**					
Symptom/problems	82.30±11.78	79.79/84.81			
Effect of kidney disease	59.59±21.70	55.28/64.53			
Burden of kidney disease	55.24±26.32	49.63/60.85			
Work status	22.41±13.89	15.19/29.63			
Quality of social interaction	82.60±15.43	79.31/85.89			
Cognitive function	83.67±15.90	80.28/87.06			
Sexual function	64.79±30.05	58.39/71.20			
Sleep	72.84±19.65	68.65/77.03			
Social support	94.35±12.53	91.68/97.02			
Dialysis staff encouragement	99.13±4.58	98.16/100.11			
Patient satisfaction	97.45±8.41	95.66/99.25			
**SF-36 domain/component summary score**					
Physical functioning	47.91±27.17	43.10/52.72	252.36±135.20	113.31/393.04	p<0.001*
Role-Physical	36.80±33.48	29.10/44.49	63.18±42.99	51.55/74.80	p<0.001*
Role-Emotional	57.60±44.67	49.69/65.50	65.45±45.34	53.19/77.71	p=0.125
Bodily pain	70.34±28.97	65.21/75.46	65.13±26.01	58.10/72.16	p=0.031*
General health	53.00±22.54	49.00/56.99	60.63±18.78	55.55/65.71	p=0.176
Mental Health	74.59±18.52	71.31/77.87	67.34±18.29	62.39/72.29	p=0.459
Social Functioning	67.00±26.48	62.31/71.68	70.90±22.70	64.77/77.04	p=0.684
Vitality	56.96±24.23	52.66±61.25	56.27±19.63	50.96/61.57	p=0.714
PCS	52.01±21.82	48.12/55.87	110.32±34.33	18.05/202.60	p=0.028*
MCS	64.03±65.37	60.20/67.87	64.99±21.59	59.15/70.83	p=0.678

Note: The data are presented as means ± standard deviations (SD) for each domain or component, along with 95% confidence intervals (CI). This table compares health-related quality of life (HRQoL) between patients with stage G5 chronic kidney disease treated with dialysis (CKD G5D) and healthy controls. HRQoL was assessed using the Kidney Disease Quality of Life Short Form (KDQOL-SF), which includes disease-targeted scales (such as symptom burden, work status, and cognitive and sexual function) as well as general health domains derived from the Short Form-36 (SF-36) instrument. The SF-36 measures physical and mental health across multiple domains and generates two composite scores: the Physical Component Summary (PCS) and the Mental Component Summary (MCS). Statistically significant differences ($p < 0.05$) indicate that CKD G5D patients experience a lower quality of life in several domains compared to healthy individuals.

*Significant at the level of $p<0.05$.
